# Effects of repeated sprints training on fracture risk-associated miRNA

**DOI:** 10.18632/oncotarget.24707

**Published:** 2018-04-06

**Authors:** Veronica Sansoni, Silvia Perego, Gianluca Vernillo, Andrea Barbuti, Giampiero Merati, Antonio La Torre, Giuseppe Banfi, Giovanni Lombardi

**Affiliations:** ^1^ Laboratory of Experimental Biochemistry and Molecular Biology, I.R.C.C.S. Istituto Ortopedico Galeazzi, Milan, Italy; ^2^ Human Performance Laboratory, Faculty of Kinesiology, University of Calgary, Calgary, Canada; ^3^ Department of Biosciences, Università degli Studi di Milano, Milan, Italy; ^4^ Centro Interuniversitario di Medicina Molecolare e Biofisica Applicata, Università degli Studi di Milano, Milan, Italy; ^5^ Department of Biomedical Sciences for Health, Università degli Studi di Milano, Milan, Italy; ^6^ IRCCS Fondazione Don Carlo Gnocchi, Milan, Italy; ^7^ Università Vita-Salute San Raffaele, Milan, Italy

**Keywords:** repeated-sprint training, fracture risk, miRNA, bone markers, real-time PCR

## Abstract

Repeated-sprint training (RS, short-duration sprints at supramaximal intensities interspersed with brief recoveries) is a time-saving metabolically effective strategy whose effects on bone are unknown. Bone metabolism is a finely regulated process profoundly affected by exercise as assayable by studying specific systemic (e.g., hormones, cytokines) and bone-derived molecules (e.g., bone markers, miRNAs). Aim of this study was to determine the effect of a 8-week repeated-sprint on circulating levels of fracture risk-associated miRNA. Blood was collected from 9 subjects performing RS 3 times/week (EXP) and 9 age-matched inactive controls (CTRL) before the start of the protocol (T0) and after 4 (T1) and 8 weeks (T2). The relative expression of miR-21-5p, miR-23a-3p, miR-24-3p, miR93-5p, miR-100-5p, miR-122-5p, miR-124-3p, miR-125b-5p, miR-148a-3p, miR-637 was assayed by real-time PCR by the 2**^−ΔΔCT^** method (housekeeping: miR-425-5p, miR-484). Serum concentrations of bone markers (DKK1, sclerostin, osteoprotegerin, osteocalcin, osteopontin), cytokines (IL-1β, TNFα), and metabolic hormones (leptin, insulin, PTH) were assayed by multiplex assay. miR-637 and miR-124-3p were undetectable. In CTRL miRNA levels remained unchanged. In EXP miR-21-5p remained unchanged. Compared to T0 miR-23a-3p and miR-24-3p were significantly decreased at T1 and T2, also compared to CTRL, miR-100 was significantly decreased at T2, miR-122-5p, miR-125-5p, and miR148a-3p were significantly decreased at T1, while miR-93-5p was significantly increased at T1. None of the metabolic hormones was affected by the intervention while, among the bone markers, DKK1, osteocalcin and sclerostin were slightly but significantly decreased. In conclusion, an 8-week repeated-sprint training downregulates the expression of circulating miRNA associated with fracture risk.

## INTRODUCTION

Among the many factors influencing morbidity and mortality with increasing age, maintenance of healthy bone is an important variable for general health and quality of life. This is especially true since bone exerts important endocrine functions, besides the classical ones (e.g., locomotor, protection, mineral reservoir, hematopoiesis) [1–4]. Indeed, the bone involvement within the energy metabolism is evidenced in several physiological (e.g., exercise) and pathological (e.g., metabolic syndrome, diabetes) conditions in which by affecting bone metabolism impacts on energy metabolism and *vice versa* [5, 6].

Osteoporosis is a worldwide health problem [7] and physical exercise is a valid and effective to prevent or counteract osteoporosis in all life stages in both women and men [8, 9].

Sprint running, as well as endurance running which is known to improve BMD, are both weight-bearing activities. The differential bone-related effects result mainly from differences in energy substrate utilization and specific metabolic adaptations [10]. Repeated-sprint (RS), characterized by short-duration sprints at supramaximal intensity interspersed with brief recovery (interval training), has been proposed as a time-efficient strategy and effective and viable alternative to endurance training protocols [11, 12]. As for other kinds of interval trainings, encouragement to RS training in healthy and diseased people is supported by several social and psychological reasons [13]. First, being remarkably time efficient, it fits better with Western society's time-poor lifestyle. Second, it may be more attractive for individuals (e.g., sedentary) who difficulty handle exercise sessions perceived monotonous and too long. Third, RS training results in the maintenance of and improvement in muscular strength since it requires a high level of muscular power. As such, it is considered a reliable and time-efficient strategy to stimulate metabolic adaptations in skeletal muscles [13].

The scientific interest around the interval training is growing due to the evidences about its benefits in several pathological conditions, besides the well-known benefits of physical activity in general. High-intensity training programs have been demonstrated to be beneficial in 25 medical conditions, including cardiovascular disease, stroke, hypertension, cancer, and type 2 diabetes [14]. Interestingly, beside the possible preventive effects of physical activity, and particularly of interval training, in cancer onset, this kind of physical activity has been indicated as very effective in improving life quality in tumor survivors and to reduce the fatigue sensation following chemotherapy [15].

Among the interval training typology, RS training has been shown to exert multiple beneficial effects in healthy and diseased human subjects, including improved cardiorespiratory fitness, glycemic control [16], heart rate [17], and adiposity [18]. However, no information are still available about the effect of RS training on bone metabolism.

Classical and last-generation biochemical markers of bone turnover are exceptional tools for monitoring the effects of exercise protocols on metabolism and endocrine function of bone. They give an instantaneous picture of acute or established metabolic responses of the bone to a specific stimulus, before the architectural modifications ascertainable through radiographic techniques (e.g., dual-energy X-ray absorptiometry [DXA]) [19]. However, the stimulus should exceed a threshold in intensity and duration before changes in circulating marker concentration can be appreciated. Changes in bone turnover markers may be preceded by changes in even more sensitive markers, such as microRNAs (miRNAs) [20, 21].

miRNAs are short (about 18 nucleotides) endogenous non-coding RNAs that regulate gene expression post-transcriptionally. They target complementary mRNAs and induce their degradation. A single miRNA affects the expression of hundreds of genes [22]. miRNAs are also released into the circulation and their level changes with the multiple biologic adaptive responses to specific environmental conditions. Hence, circulating miRNAs provide a useful tool for studying the biological effects of interventions such as exercise and diet [23].

Several miRNAs regulate osteoblastogenesis and osteoclastogenesis by modulating the expression of key factors regulating these two processes and many of them have the potential for use as biomarkers of bone health status or therapy monitoring [24]. Indeed, altered expression of specific miRNAs has been associated with fracture risk and metabolic disorders of bone [20, 21]. This knowledge could be applied to diagnostic and therapeutic purposes [25].

However, the knowledge of the modulatory effects of exercise protocols on miRNAs associated with bone turnover and fracture risk is still very limited. This connection, although theoretically obvious, has been only suggested, or even speculated, but, to our knowledge, it has never been tested [26, 27]. This is of particular interest since, being miRNA biomarkers of early response, once clearly depicted their physiology, they would be applied in routine settings to verify the response to a specific intervention. Based on this background, with this study we aimed at determining the effects of an 8-week RS training protocol in healthy, young physically active males on the expression profile of selected fracture-risk-associated miRNAs, as well as on bone and energy metabolism markers, as compared to age-matched controls.

## RESULTS

**Figure 1:**
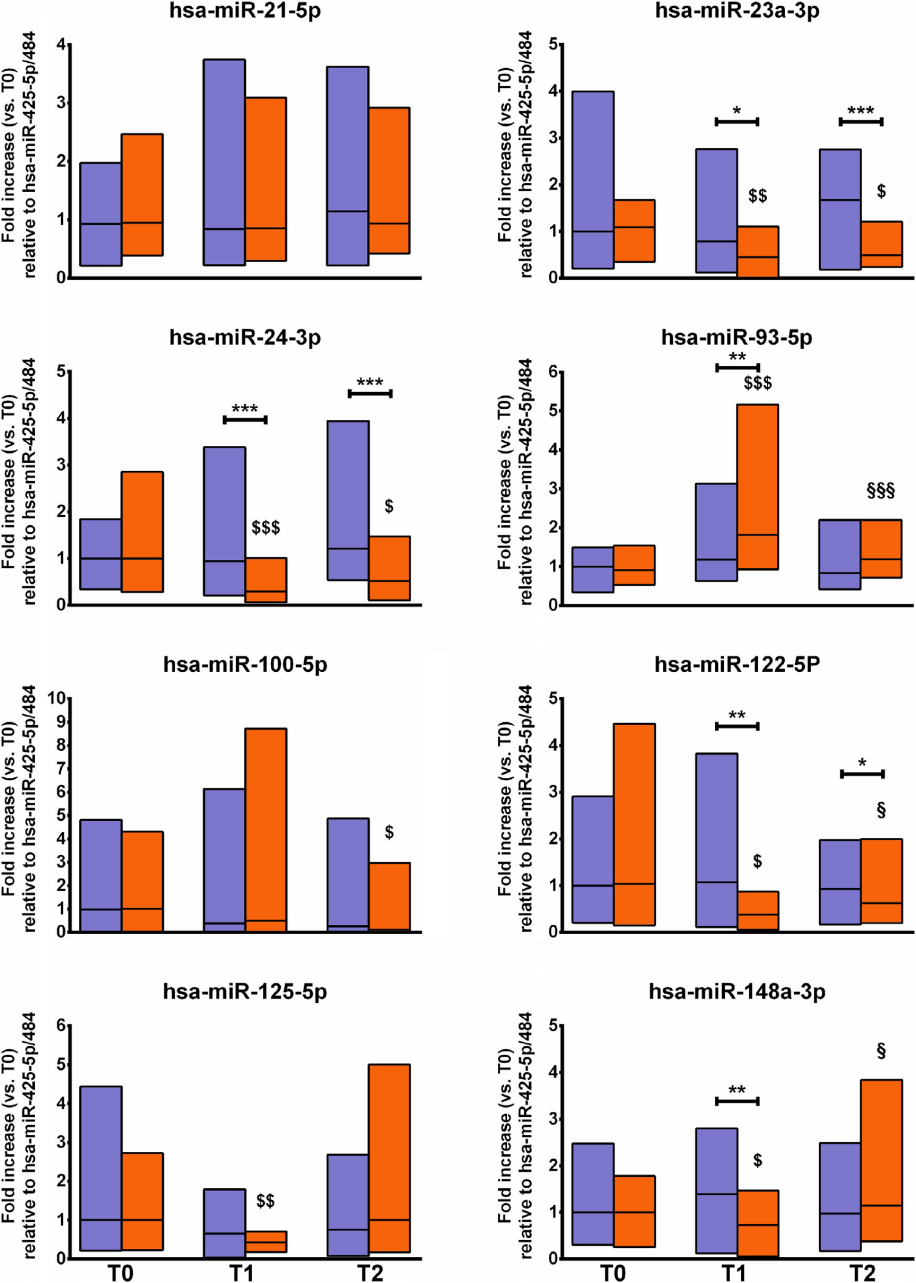
Changes in fracture risk-associated circulating miRNAs The figure shows the changes in the profile of circulating miRNA associated with fracture risk before (T0), during (T1), and after (T2) an 8-week repeated-sprints training protocol in the experimental group (orange blocks) and the controls (violet blocks). The relative expression of target miRNA is normalized to miR-425-5p and miR-484, here considered as housekeeping. The mean T0 values of the CTRL group were set at 1. Blocks indicate the ranges, central lines indicate the mean values. Asterisks indicate significant intergroup differences at a specific time point (^*^p < 0.05; ^**^p < 0.01; ^***^p < 0.001). Symbols on bars indicate within-group difference vs. T0 (^$^*p <* 0.05; ^$$^*p <* 0.01; ^$$$^*p <* 0.001) and vs. T1 (^§^*p <* 0.05; ^§§^*p <* 0.01; ^§§§^*p <* 0.001).

Figure 1 presents the changes in the panel of miRNAs assayed. These are the main findings: i) miR-21-5p was stable; ii) miR-23a-3p and miR-24-3p were decreased at T1 and T2 compared to T0 and to CTRL; iii) miR-100 was decreased at T2 compared to T0; iv) miR-122-5p, miR-125-5p, and miR-148-3p were decreased at T1 and then recover to baseline at T2; iv) miR-93-5p was increased by the intervention.

More in detail, none of the miRNAs analyzed at T0 differed between the EXP and CTRL groups, indicating the homogeneity of the study sample. miR-124-3p and miR-637 remained undetectable in both groups during the entire study period. In EXP, miR-21-5p was neither affected by the intervention nor differed from CTRL at any time point. A similar trend was noted for miR-23a-3p and miR-24-3p: the levels were decreased between T0 and T1 by about 20% (*p* < 0.01) and 70% (*p* < 0.001), respectively, and at T2 by about 20% and 50%, respectively (*p* < 0.05 for both). Moreover, the levels were lower compared to those measured in CTRL at T1 (*p* < 0.05 and *p* < 0.001, respectively) and T2 (*p* < 0.001 for both). miR-100-5p showed the highest variability in both groups: it was slightly decreased in EXP at T2 compared to T0 (*p* < 0.05). miR-122-5p and miR-148a-3p were decreased (50% and 30%, respectively) in EXP at T1 as compared to T0 (*p* < 0.05 and *p* < 0.01, respectively) but returned to baseline at T2 (*p* < 0.05, compared to T1). miR-125-5p was decreased by half in EXP at T1 as compared to T0 (*p* < 0.01) although with no difference compared to CTRL. miR-93-5p was the only miRNA that increased in EXP, with a two-fold increase measured at T1 as compared to T0 (*p* < 0.001) and CTRL at T1 (*p* < 0.01); however, at T2 it returned to baseline (*p* < 0.001 compared to T1).

**Figure 2:**
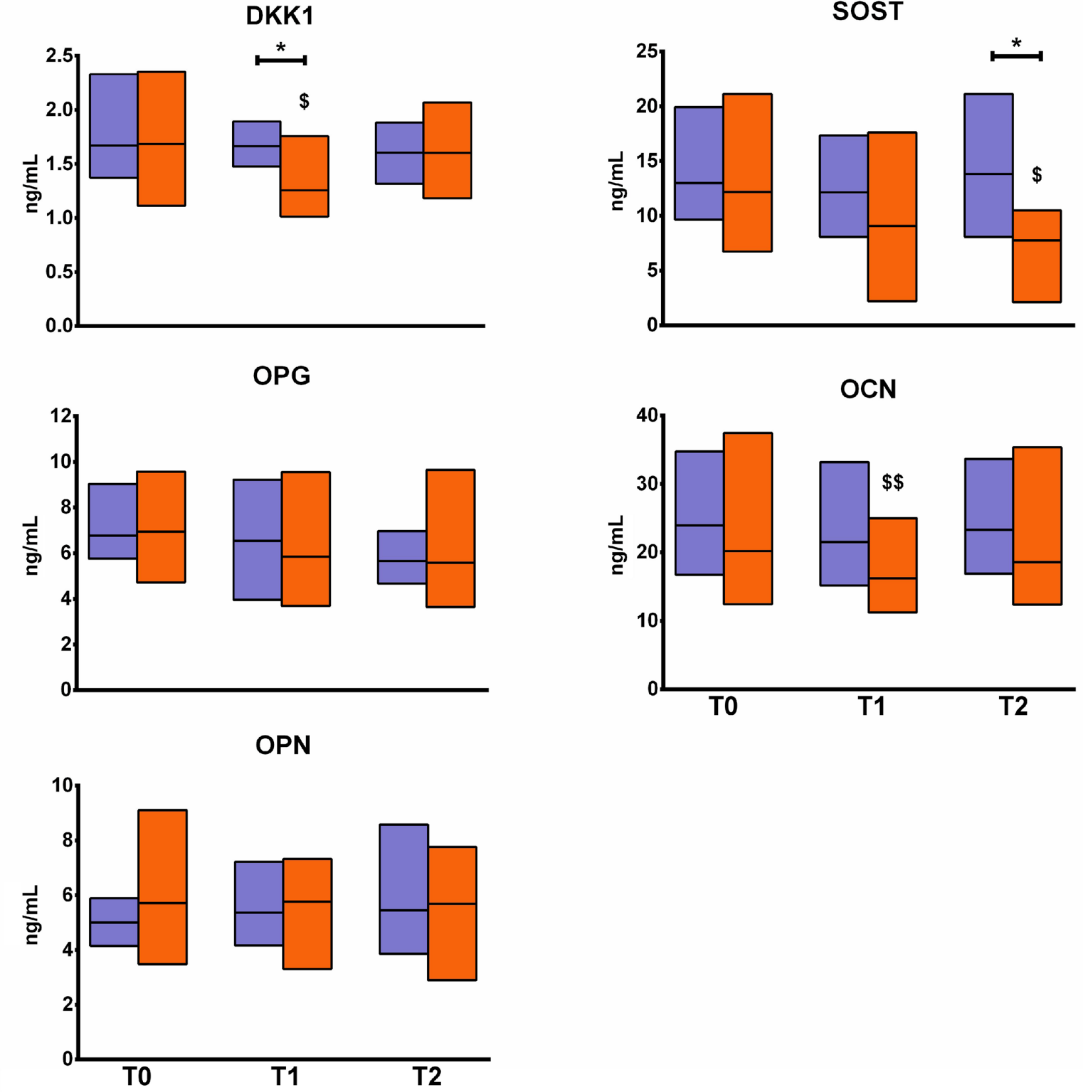
Changes in metabolic markers of bone turnover The figure shows the changes in the profile of serum levels of metabolic markers of bone turnover in the experimental group (orange blocks) and the controls (violet blocks). Blocks indicate the ranges, central lines indicate the mean values. Asterisks indicate significant intergroup differences at a specific time point (^*^p < 0.05). Symbols on bars indicate within-group difference vs. T0 (^$^*p <* 0.05; ^$$^*p <* 0.01). DKK1: Dikkopf 1; SOST: sclerostin; OPG: osteoprotegerin; OCN: osteocalcin; OPN: osteopontin.

**Figure 3:**
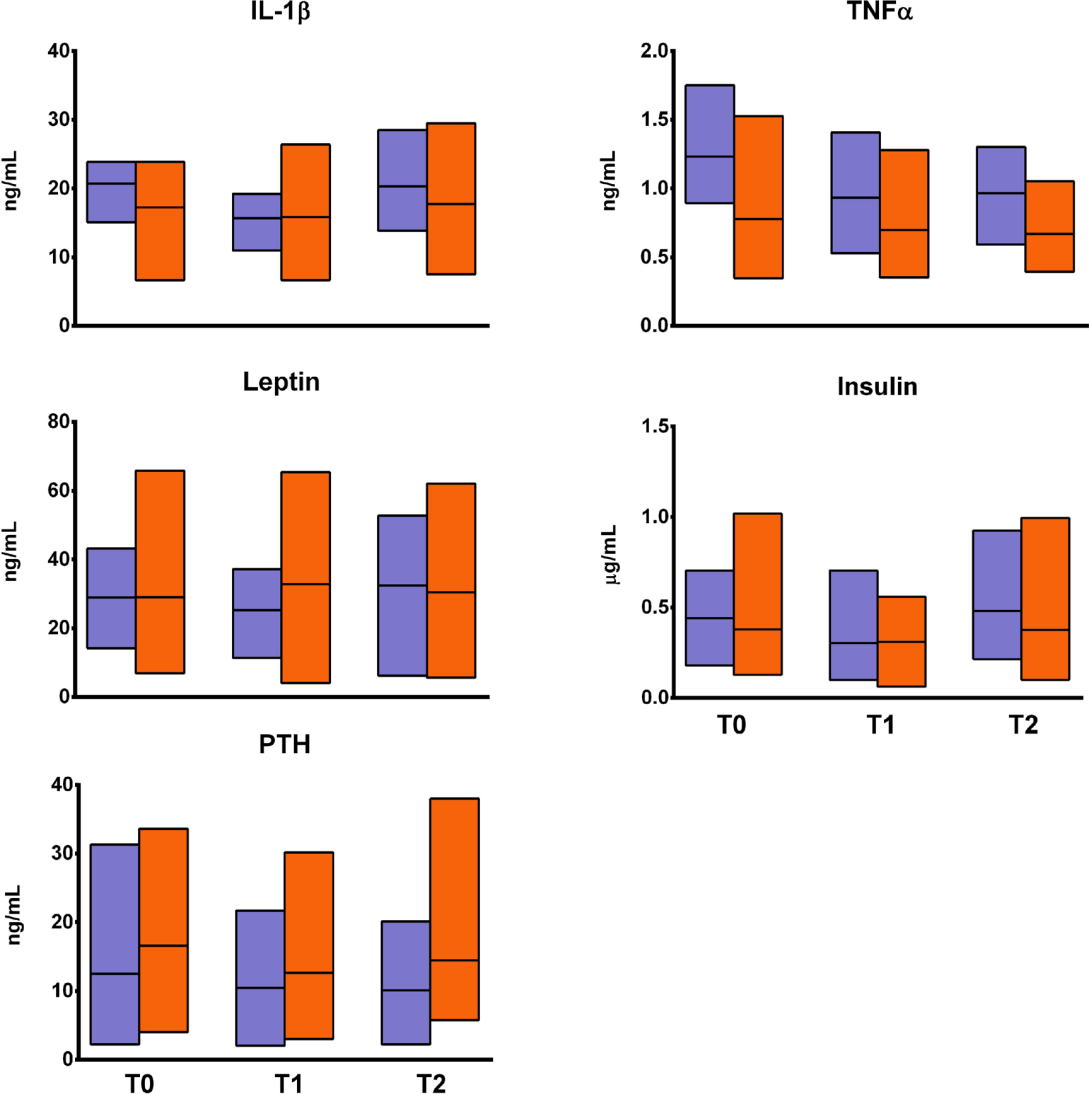
Changes in fracture inflammatory cytokines and bone metabolism-associated hormones The figure shows the changes in the profile of serum levels of cytokines (interleukin (IL)-1β, tumor necrosis factor (TNF)α), and hormones (leptin, insulin, parathyroid hormone [PTH]) associated with bone metabolism and fracture risk in the experimental group (orange blocks) and the controls (violet blocks). Blocks indicate the range, central lines indicate the mean values. None of the comparisons was found to be statistically significant.

No changes in either biochemical marker of bone turnover, OPN and OPG, were observed (Figure 2). DKK1 was significantly decreased by one fourth in EXP at T1 as compared to T0 (*p* < 0.05) but returned to baseline value at T2. DKK1 at T1was also lower in EXP than in CTRL (*p* < 0.05), as seen for OCN, which was significantly decreased in EXP at T1 (*p* < 0.01), although the difference was not significant compared to CTRL. SOST, instead, was significantly decreased in EXP at T2 as compared to T0, and also as compared to CTRL at the same time point (*p* < 0.05). No changes in inflammatory cytokines and bone metabolism-regulating hormones were observed in EXP (Figure 3).

In EXP, SOST changes were significantly, although slightly, inversely correlated with miR-93-5p and miR-122-5p (*r* = −0.38, *r* = −0.35, respectively; *p* < 0.05) as well as OPG with miR-100-5p and miR-122-5p (*r* = −0.38, *r* = −0.34, respectively; *p* < 0.05). OPG and miR-122-5p were also correlated, although directly, in CTRL (*r* = 0.71; *p* < 0.05). In CTRL, OCN was directly correlated with miR-93-5p (*r* = 0.40; *p* < 0.05), miR-100-5p (*r* = 0.39; *p* < 0.05), miR-125-5p (*r* = 0.47; *p* < 0.01), miR-148a-3p (*r* = 0.62; *p* < 0.001),but these trends were lost in EXP.

## DISCUSSION

We investigated the effect of an RS training program on the profile of a panel of free circulating miRNAs whose levels have been associated with increased risk of bone fracture. Our results show that an 8-week RS training protocol was able to modify the levels of circulating miRNA associated with fracture risk. The kinetics of response differed: miR-23a-3p, miR-24-3p, and miR-100-5p showed a net decrease by the end of the 8 weeks, while other miRNAs were only transiently modified (i.e., miR-122-5p, miR-125-5p, miR-148a-3p, miR-93-5p). Moreover, contrary to previous findings on cohorts of elderly subjects, some miRNAs associated with bone cell function, i.e., miR-124-3p [28–30] and miR-637 [25, 31, 32] were undetectable in our population.

The metabolic markers of bone turnover were only minimally affected, and only those responsive to load (e.g., DKK1, SOST, and OC) showed a response. Comparably, the set of cytokines and endocrine markers, which are considered highly sensitive to exercise interventions [6], were completely unaffected.

The selected panel of miRNAs comprises a series of miRNAs involved in osteoblastogenesis and/or osteoclastogensis, in bone cell function, and/or clinically relevant because associated with BMD and bone fracture risk. Seeliger *et al*. [20] identified specific miRNAs differentially expressed in patients with osteoporotic vs. non-osteoporotic fractures. Nine miRNAs (i.e., miR-21, miR-23a, miR-24, miR-93, miR-100, miR-122a, miR-124a, miR-125b, and miR-148a) were upregulated in the serum of patients with osteoporosis and 6 miRNAs (i.e., miR-21, miR-23a, miR-24, miR-25, miR-100, and miR-125b) were upregulated in the bone tissue of osteoporotic patients. Among the 5 miRNAs (miR-21, miR-23a, miR-24, miR-100, and miR-125b) upregulated in both serum and the bone tissue, Panach *et al.* [33] identified miR-122a-5p, mir-125b-5p, and miR-21-5p as markers for osteoporotic fractures. Given that they are clinically relevant in cohorts of elderly subjects, modulating their expression earlier in life via specific physical activity protocols, for example, could be an effective strategy for improving bone health [8]. From a health prevention perspective, RS training might offer an acceptable form of time-saving and high-intensity physical activity.

miR-21-5p, miR-122-5p, and miR-125p have been positively associated with osteoporotic bone fractures and serum cross-laps concentrations (CTx-I), independent of age [33]. Recently, miR-24-3p, one of the most affected miRNAs in our setting, was found to be highly negatively associated with BMD in osteoporotic patients, together with miR-21-5p, miR-93-5p, miR-100-5p, and miR-125-5p [34]. Indeed, miR-24 and miR-23a, within the miR-23a-24-27a cluster, inhibit osteoblast differentiation by inhibiting Runx2 via SATB2 inhibition [35]. Moreover, miR-23a-3p and miR-24-3p inhibit osteogenic differentiation by targeting Runx2 and CXCL-12 [36] and Tcf-1 [37], respectively. Yavropoulou *et al*. [38], however, recently reported an inverse association between miR-23a (and miR-21-5p, miR-124-3p) and osteoporosis severity in post-menopausal osteopenic subjects and osteoporotic patients with at least one vertebral fracture.

miR-100, another miRNA that displayed differential behavior between the EXP and the CTRL groups, is known to be involved in BMP-dependent mechanisms involved in the fate of mesenchymal stem cells towards osteoblastogenesis [39, 40]. Finally, among the miRNAs in which a decrease was noted for the EXP group, miR-148a-3p was found to be involved in hedgehog signaling as an inhibitor of osteoblast differentiation of mesenchymal stem cells [41]. miR-93, instead, which was seen to increase during the first half of the 8-week study, other than being negatively associated with BMD [34] has also been associated with inhibited osteoblastogenesis in humans and mice [42, 43].

The mechanism by which changes in circulating levels of fracture risk-associated miRNA occur after RS training is unknown. However, based on the changes in protein marker concentrations (i.e., DKK1 and SOST), we may speculate that direct biomechanical stimulation (i.e., ground reaction force plus muscle traction) might be the main one. Indeed, DKK1 and SOST are the effectors of the mechanosensitive function of bone tissue: their decrease during RS training indicates an increasing load [44, 45]. On the contrary, it seems rather unlikely that the changes in these miRNAs are dependent upon metabolic or inflammatory changes, since we did not observe any changes in the relative markers. Only miR-21 was listed in a proposed panel of mechanosensitive miRNAs [46]. The correlation analysis indicates an inverse interaction between miR-93-5p/miR-122-5p and sclerostin as well as between miR-100-5p/miR-122-5p and OPG suggesting a possible role for these miRNAs in mechanosensitity and osteoimmune crosstalk. Noteworthy, the direct association between OPG and miR-122-5p as well as the direct correlation between OCN and miR-93-5p, miR-100-5p, miR-125-5p, and miR-148a-3p only in CTRL suggest that the possible underlying regulatory mechanisms acting under resting conditions, are instead uncoupled by RS training.

The mechanosensitivity of miRNA is a fresh research topic, and further studies are still needed. Nonetheless, mechanosensitive involvement of at least some of the miRNA studied here cannot be excluded, however this hypothesis has not still been tested. Indeed, of the assayed miRNA none have been previously specifically associated with the serum levels of bone markers. Contrarily, all of them have been associated with either inflammatory (IL-1β, TNFα) or metabolic (leptin, insulin) markers although in specific pathological conditions [47].

A specific miRNA signature in osteoporosis and fracture risk is desirable, since it would open the way to developing novel diagnostic/prognostic biomarkers and support existing diagnostic tools, DXA, and bone turnover markers. This is desirable since both approaches have considerable limitations [19, 48–51]. The signatures are, at least in part, known for cardiac and neoplastic diseases [52] but not for metabolic diseases of bone. Given the putative association of circulating miRNAs with fracture risk, we thought it was interesting to study their response to a specific training protocol. This is because a study on females recently demonstrated that a school-based high-intensity physical activity represents an effective strategy for both improving peak bone mass and preventing age-dependent bone loss [8].

In recent literature, studies on the effects of different physical activity protocols on the circulating levels of different panel set of miRNAs are abundant, as reviewed in [53, 54]. The short-term, or even acute, response of miRNA to exercise has been highlighted by previous studies, thus supporting our findings, although on different sets of miRNA. Eight-week-long explosive strength training (EXPL), hypertrophic strength training (HYP), and high-intensity interval training (HIIT) in trained subjects, for example, had similar inhibitory effects on four miRNAs (miR-16, miR-21, miR-93 and miR-222) and the baseline levels of miR-93 independently predicted the isometric extension of the leg [55]. In an experimental setting similar to ours, 4-to-6 sprints, 3 times per week over 6 weeks decreased the circulating levels of 4 miRNA associated with muscle development (myo-miRNA: miR-1, miR-133a, miR-133b, miR-486) [56]. Very recently, the miRNA response following an half-marathon was studied, with a particular focus on miR-133 and miR-206 that increased parallel with the increase of the biomarkers of nonspecific tissue damage [57].

The circulating miRNA profile reflects intracellular miRNA expression and can serve as marker of the biological processes taking place in cells. Owing to their place in the hierarchical process leading to gene expression just before the translation of the genetic code into a protein, as fine-tune modulators, the information deriving from their circulating levels may eventually predict an early biological response to a specific condition [23, 58].

A possible limitation is that, although the levels of the circulating miRNAs selected for this study have been associated with fracture risk, they do not necessarily mark a bone metabolic process since, under the experimental conditions analyzed here, they could be produced by many other cell types. However, despite the multiple targets of each single miRNA, this study evidences that some of those miRNAs whose high circulating levels have been associated with an increased fracture risk are actually reduced by a specific protocol of physical activity. It is plausible that RS training has positive effects on bone metabolism, although the current setting did not allow us to investigate the role of intermittence. Furthermore, our study demonstrated that miRNAs, as key regulators of cellular functions, respond to stimuli much more sensibly than classical biochemical markers, and that circulating miRNAs may mark an early response to a physiological or pathological stimulus [23]. However, the lack of any previous information about the association between the assayed miRNA and the bone markers here considered should be highlighted. Hence, either a direct inter-dependency or a co-consequence of the same stimulus might be hypothesized.

To this end, the study of the physiological response of miRNAs, particularly to exercise protocols, holds importance because it may help to improve current knowledge and limit our uncertainty about the physiological role of these mediators/markers. To our knowledge, this is the first study to explain the modifications in the circulating levels of a panel of fracture-risk associated miRNAs to a specific exercise protocol.

In conclusion, our findings demonstrate that brief, supramaximal running training in young males can reduce the circulating levels of some of the miRNA that have been associated with fracture risk in elderly populations. This supports the hypothesis for a potential preventive role of such exercise training against osteoporosis.

## MATERIALS AND METHODS

### Participants

This randomized, controlled longitudinal experimental protocol was part of a larger study investigating cardiovascular, metabolic, inflammatory, and bony adaptations after a RS training protocol [17]. In accordance with the Declaration of Helsinki, all subjects were informed about the procedures, benefits, and possible adverse events associated with the protocol and they gave their written, consent. The protocol was approved by the Comitato EticoOspedale San Raffaele (Milan, Italy).

The study cohort consisted of 18 healthy, physically active male adults. Sample size was calculated based on the requirements of the previous study [17]. Inclusion criteria were: no history or current clinical signs of cardiovascular, pulmonary, metabolic or bone diseases; no traumatic fractures in the 2 previous years; no routine involvement in intermittent sports (e.g., soccer, handball, basketball); physical activity below the moderate level according to American College of Sports Medicine (ACSM) guidelines (i.e., a minimum of 30 min of aerobic activity, 5 times a week [59]). Participants were randomly assigned to the experimental group (EXP) that performed RS training or the control group (CTRL) that performed normal, daily physical activities, as described in [17]. The two groups were comparable for anthropometric characteristics (Table 1).

**Table 1:** Anthropometric features of the study population

	EXP (*n* = 9)	CTRL (*n* = 9)
**Age (years)**	24.3 ± 3.7	23.8 ± 4.4
**Height (m)**	1.78 ± 0.07	1.83 ± 0.07
**Body mass (kg)**	69.9 ± 5.8	73.0 ± 7.2
**BMI (kg/m^2^)**	22.1 ± 1.7	21.8 ± 1.1

Data are expressed as mean ± SD. BMI: body mass index.

### Experimental protocol

#### Test session

A test session was performed 1 week before the beginning of RS training to familiarize the subjects with the experimental protocol. Testing, as well as the following experimental phase, was performed on a synthetic indoor track at a constant temperature of 18–20° C. The test entailed five 30 m sprints interspersed by 25 s of active recovery [17, 60]. Before each test session, the subjects completed a 10 min warm-up of low-intensity running and striding, followed by three submaximal 30 m sprints with 120 s of active recovery that served to (re-)accustom them to the testing procedures. Then, each subject completed a preliminary, single 30 m sprint test. This trial served to avoid adoption of protective pacing strategy and to have a criterion score for the subsequent 5 × 30 m sprint test. Specifically, in case the performance time for the first sprint of the RS test was > 2.5% than the criterion score, the test was terminated and the RS test had to be repeated, after a 5 min rest, with greater effort [17, 61]. Five seconds before starting each sprint, the participants assumed the standardized ready position [62], and waited for the start signal from the countdown given by the researcher.

The subjects were asked to maintain their usual eating habits throughout the duration of the protocol, but were told to refrain from caffeine and alcohol intake during the 12 h preceding a test session.

#### RS exercise

**Figure 4:**
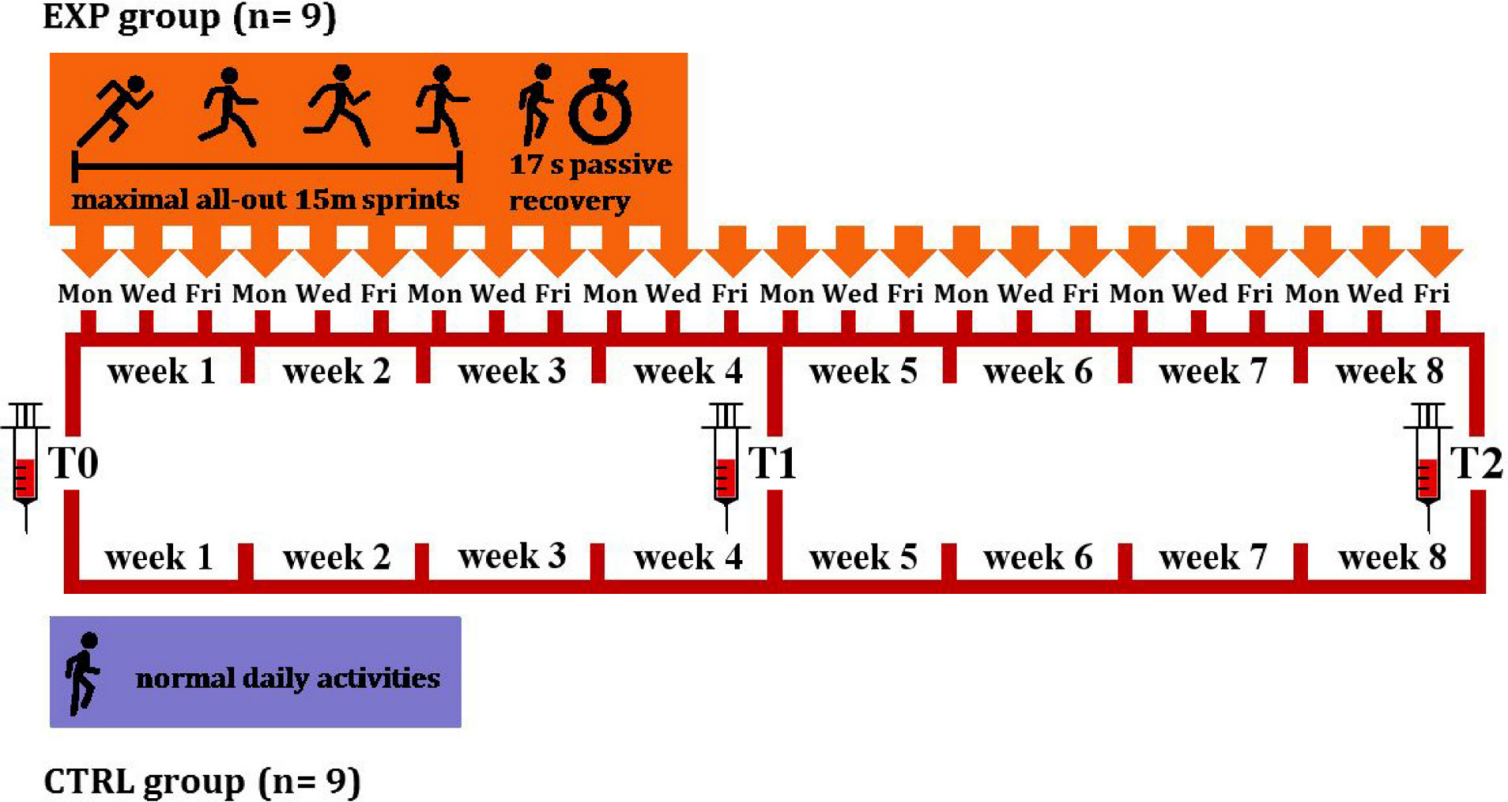
Experimental protocol scheme 18 subjects were randomly assigned to either an experimental (EXP, orange) or a control (CTRL, violet) group. EXP (*n* = 9) performed 18 maximal all-out 15 m sprints with 17 s of passive recovery, 3 times/week, over 8 weeks. CTRL (*n* = 9) carried out normal daily activities for 10 weeks. Blood was sampled in both groups before the start of the protocol (T0), at 4 weeks (T1), and then the week after completion of the 8 week training period (T2).

RS exercise consisted of 18-times repeated 15 m sprints with 17 s of passive recovery [13, 17], after each bout the subjects ran in the opposite direction. Each set of exercise, lasting 6 min, was repeated 3 times a week, with at least a 48 h recovery between each test. Before each training session, a 10 min warm-up of low-intensity running and striding, was performed by the subjects, followed by 3 submaximal 15 m sprints with 60 s of passive recovery that served to (re-)accustom them to the experimental procedures. The protocol lasted 8 weeks and a total of 24 RS sessions were performed by each subject of the EXP group. A schematic illustration of the protocol is shown in Figure 4.

### Blood sampling and testing

#### Blood sampling

Blood samples were taken from all subjects at baseline, before the start of the protocol (T0), after the end of the 4th week (after the 12th RS session, T1), and the day after the end of the protocol (after the end of the 8th week (i.e., after the 24th RS session) T2). Blood was collected in the morning after overnight fasting, between 8:00 and 9:00 by standard antecubital venipuncture in K2EDTA spray-coated (Vacutainer, Becton Dickinson & Co., Franklin Lakes, NJ, USA) and serum SSTII Advance (Becton Dickinson & Co.) tubes. After collection, the tubes were inverted at least 10 times (EDTA tubes were also homogenized for 15 min), stored upright at 4° C, and brought to the laboratory. Plasma and serum were separated by centrifuging whole blood at 1300 *g* for 10 min at 15° C and then aliquoted in plain apyrogen DNase/RNase-free tubes (Eppendorf AG, Hamburg, Germany). Plasma aliquots for miRNA analyses were further centrifuged at 300 *g* for 5 min at room temperature (RT, 22° C). The supernatant was transferred to a new plain tube and stored at −80° C until assayed. The pre-analytical phase strictly adhered to standard [2] and circulating miRNA-specific [23] precautions.

### Circulating miRNA analyses

MicroRNA-enriched total RNA was extracted from plasma with a miRCURY™ RNA Isolation Kit (Exiqon A/S, Denmark). Extraction efficiency was checked through 3 synthetic oligonucleotides (spike-ins: Sp2, Sp4, Sp5) added at recommended concentrations. Reverse transcription and real-time PCR were performed with a miRCURY LNA™ Universal RT microRNA PCR and Universal cDNA synthesis kit II (Exiqon), using Sp6 and Cel39 as internal controls. Real-time PCR was performed using specific miRCURY LNA™ microRNA PCR primers (Exiqon; Supplementary Table 1). The following panel of fracture risk-associated circulating miRNAs was tested: miR-21-5p, miR-23a-3p, miR-24-3p, miR93-5p, miR-100-5p, miR-122-5p, miR-124-3p, miR-125b-5p, miR-148a-3p, miR-637. Relative expressions were calculated by the 2^–ΔΔCT^ method. miR-425-5p and miR-484 were chosen as housekeeping since they result to be more stable in this setting. Hemolysis was checked by calculating the miR-23a-to-miR-451 ΔCT ratio: a value greater than 7 was considered as indicative of hemolysis [63].

### Biochemical markers analyses

A panel of biochemical markers associated with either bone or energy metabolism was measured in serum to test for possible metabolic established effects of the training protocol. The biomarkers were tested through a Milliplex^®^ multiplex immuno-based fluorescent Luminex ^®^assay (EMD Millipore, Billerica, MA, USA) on a Luminex MagPix^®^ system (Bio-Rad Laboratories, Hercules, CA, USA). Briefly, the assay allows the measurement of multiple analytes within the same well, containing the same sample aliquot through the addition of magnetic fluorescent beads coated with antibodies against the specific analyte of interest, thus completely avoiding inter-assay variability. The analytes, and the relative intra- (CV_w_) and inter-assay (CV_b_) variability, were: interleukin (IL)-1β (7%, 9%), tumor necrosis factor (TNF)-α (8%, 7%), DKK-1, sclerostin (SOST; 6%, 13%), osteocalcin (OCN; 5%, 12%), osteopontin (OPN; 2%, 12%), osteoprotegerin (OPG; 5%, 11%), parathyroid hormone (PTH; 4%, 9%), insulin (6%, 8%), and leptin (5%, 11%).

### Statistical analysis

Based on their parametric distribution (D’Agostino-Pearson omnibus normality test), the parameters are described as mean ± SD and range. For miRNA analysis, T0 values for CTRL were set at 1 and used for normalization. Within-group time-dependent modifications were analyzed by repeated one-way ANOVA for paired samples and *post hoc* Bonferroni's test. Between-group differences, at each time point, were analyzed with the unpaired *t* test.

The correlations between miRNA and bone markers trends over the time was tested with the Spearman's rank correlation test.

Statistical analysis was performed using Prism v6.01 (GraphPad Software, Inc., La Jolla, CA, USA). Comparisons were considered statistically significant when *p* < 0.05.

## SUPPLEMENTARY MATERIALS AND TABLES



## Abbreviations

miRNA: micro RNA
RS: repeated sprint
EXP: experimental group
CTRL: control group
IL-1β: interleukin
TNFα: tumor necrosis factor α
PTH: parathyroid hormone
BMD: bone mineral density
DXA: dual-energy X-ray absorptiometry
OPN: osteopontin
OPG: osteoprotegerin
OCN: osteocalcin
SOST: sclerostin
ACSM: American College of Sports Medicine
K2EDTA: ethylendiaminotetraacetate dipotassium salt
CV_w_: intra-assay variability
CV_b_: inter-assay variability.
