# Supplementary material for: Effects of repeated sprints training on fracture risk-associated miRNA
Source: Oncotarget. 2018 Apr 6;9(26):18029–40. doi: 10.18632/oncotarget.24707 (PMC5915055; doi:10.18632/oncotarget.24707)
Supplement: Supplementary file 1 [file oncotarget-09-18029-s001.pdf]

Effects of repeated sprints training on fracture risk-associated miRNA

SUPPLEMENTARY MATERIALS

Supplementary Table 1: miRNA identification for primers search

miRNA ID	miRBase Accession number	miRBase Mature Sequence Accession Number	Sequence	Exiqon code
hsa-miR-21-5p	MI0000077	MIMAT0000076	UAGCUUAUCAGACUGAUGUUGA	EX204230
hsa-miR-23a-3p	MI0000079	MIMAT0000078	AUCACAUUGCCAGGGAUUUCC	EX204772
hsa-miR-24-3p	MI0000080	MIMAT0000080	UGGCUCAGUUCAGCAGGAACAG	EX204260
hsa-miR-93-5p	MI0000095	MIMAT0000093	CAAAGUGCUGUUCGUGCAGGUAG	EX204715
hsa-miR-100-5p	MI0000102	MIMAT0000098	AACCCGUAGAUCCGAACUUGUG	EX205689
hsa-miR-122-5p	MI0000442	MIMAT0000421	UGGAGUGUGACAAUGGUGUUUG	EX205664
hsa-miR-124-3p	MI0000443	MIMAT0000422	UAAGGCACGCGGUGAAUGCC	EX206026
hsa-miR-125b-5p	MI0000446	MIMAT0000423	UCCCUGAGACCCUAACUUGUGA	EX205713
hsa-miR-148a-3p	MI0000253	MIMAT0000243	UCAGUGCACUACAGAACUUUGU	EX205867
hsa-mir-637	MI0003652	MIMAT0003307	ACUGGGGGCUUUCGGGCUCUGCGU	EX205679
hsa-miR-425-5p	MI0001448	MIMAT0003393	AAUGACACGAUCACUCCCGUUGA	EX204337
hsa-mir-484	MI0002468	MIMAT0002174	UCAGGCUCAGUCCCCUCCCGAU	EX205636
hsa-miR-451a	MI0001729	MIMAT0001631	AAACCGUUACCAUUACUGAGUU	EX204734

miRNA primers set were purchased from exiqon as describe in materials and methods.
